# How does the Nigerian health system serve people living with disabilities? A socio-ecological analysis on people living with visual impairment

**DOI:** 10.1080/16549716.2025.2581456

**Published:** 2025-12-16

**Authors:** Nneoma Dike, Lucia D’Ambruoso, Heather May Morgan, Zoë Christina Skea, Bernadine Nsa Ekpenyong

**Affiliations:** a Department of Ophthalmology, Rivers State University Teaching Hospital, Port Harcourt, Nigeria; b Institute of Applied Health Sciences, University of Aberdeen, Aberdeen, UK; c Aberdeen Centre for Health Data Science, Institute of Applied Health Sciences, School of Medicine, Medical Sciences and Nutrition, University of Aberdeen, Aberdeen, UK; d Epidemiology and Global Health, Department of Clinical Medicine, Umeå University, Umeå, Västerbotten County, Sweden; e Department of Global Surgery, Stellenbosch, University, Stellenbosch, Western Cape, South Africa; f MRC/Wits Rural Public Health and Health Transitions Research Unit (Agincourt), School of Public Health, University of the Witwatersrand, Johannesburg, South Africa; g Public Health, National Health Service (NHS) Grampian, Aberdeen, UK; h Aberdeen Centre for Evaluation (Formerly Known as Health Services Research Unit), University of Aberdeen, Aberdeen, UK; i Department of Public Health, College of Medical Sciences, University of Calabar, Calabar, Nigeria

**Keywords:** health sector, sub-saharan Africa, blindness, health, persons living with disabilities

## Abstract

People living with disabilities (PLWD) in Nigeria experience significant disparities in health outcomes and access to care. Among them, individuals living with visual impairment (VI) face compounded barriers due to structural inequities and social exclusion. This paper explores how the Nigerian health system serves PLWD through a socioecological lens, with a focus on those living with VI. We conducted a narrative review using databases such as Medline, Scopus, Embase and Google Scholar, along with policy repositories, to identify peer-reviewed and grey literature. Inclusion criteria focused on studies addressing disability, healthcare access, and Nigeria. Thematic analysis was guided by the Dahlgren and Whitehead‘s socioecological model to explore multilevel determinants affecting access to care for PLWD, particularly those living with VI. Findings reveal systemic failures across political, economic, sociocultural, and environmental domains that limit equitable access to care. Attitudinal barriers, inadequate infrastructure, and weak enforcement of anti-discrimination laws emerged as critical challenges. This analysis underscores the need for multisectoral reforms that address both institutional and societal barriers to inclusion. Strengthening legal frameworks, increasing budgetary allocations for inclusive services, and involving PLWD and people living with VI in policy processes are critical steps toward equitable healthcare delivery.

## Paper Context

- **Main findings**: The Nigerian health system is overburdened and does not equitably and adequately address the healthcare needs of people living with disabilities including visual impairment.
- **Added knowledge**: This review being the first of its kind applies the Dahlgren and Whitehead’s socioecological lens to depict the concept of reciprocal influence resulting from an overburdened health system whereby people living with disabilities and visual impairment are increasingly predisposed to communicable and non-communicable diseases.
- **Global health impact for policy and action**: Prioritise the implementation of the existing health policies and enforcement of the anti-discrimination laws, make urgent provisions to initiate regular and substantive social welfare payments to people living with disabilities and active involvement of target population at all levels in designing and implementing health policies and programmes in Nigeria to improve project outcomes.

## Background

In Nigeria, over 29 million people live with some form of disability [1], yet systemic barriers continue to exclude them from equitable access to healthcare. Among these, people living with visual impairment (VI) face compounded challenges due to physical inaccessibility, social stigma and policy neglect. This paper explores how the Nigerian health system serves people living with disabilities (PLWD), using a socioecological (SE) lens to uncover structural inequities and pathways for reform.

People’s health and wellbeing are underpinned by the performance of the health system [2]. While ensuring the highest attainable standard of population health, a health system should concurrently strive to address health inequalities [3]. Evidence shows that PLWD in Nigeria have higher predispositions to major depressive disorders, lower quality of life [4], self-esteem and health status [5]. Hence, the condition of disability introduces risks including hospitalization or other contact with the Nigerian health system [6].

In 2020, 24 million people residing in Nigeria were diagnosed with VI [7]. People living with VI experienced disproportionate risks of communicable and non-communicable diseases (NCDs) such as diabetes, depression, COVID-19, and hypertension [8,9]. Additionally, they experienced the highest levels of functional difficulty among other PLWD [10]. Taken together, people living with VI in Nigeria may have greater healthcare needs and be disproportionately impacted by inequality in access to healthcare.

The situation necessitates a deeper analysis of the Nigerian health system to understand its capacity to provide inclusive healthcare for people living with VI. Little is known about the resilience of the Nigerian public health system to address the healthcare needs of this population. Furthermore, there is limited disability-related data in Nigeria and a dearth of scientific literature on the general accessibility of Nigerian healthcare services [1,11].

Previous studies have examined health disparities among PLWD globally [12,13]. However, few have explored the layered determinants of health access in Nigeria. This paper builds on recent analyses [14,15] by incorporating structural and interpersonal factors to create a comprehensive systems picture and potential avenues for reform. Additionally, despite existing analyses of the Nigerian health system [14–17], no study to date has comprehensively reviewed the robustness of the Nigerian health system to address the healthcare needs of PLWD focusing on people living with VI. This narrative review elected to assess provisions and gaps in healthcare access for PLWD in general in Nigeria with a focus on people living with VI due to their higher exposure to risk including the possibility of living with additional disabilities [8,9].

A health system encompasses activities, individuals and agencies whose major objective is to foster, restore and preserve health [18]. However, it has been argued that the health system is more than a mechanical framework instead, it is a core social system [19–22]. Hence, a more practical definition of a health system in a society is one which maintains a homeostatic balance with the said society as well as other systems operating within this society whereby if a part of this homeostatic system malfunctions, it impacts the needs and interests of the rest of the systems [23].

It is well established that the majority of health and health inequalities are produced in the conditions in which people live and grow, and that these are outside the conventional health system [24–27]. For instance, evidence has shown that many of the barriers to equitable healthcare for people living with VI in Nigeria are rooted in the wider sociocultural and economic contexts [28,29]. This necessitated the need to apply a SE framework to this review to understand the broader societal influences. The authors appraised different ecological models as frameworks but Dahlgren and Whitehead’s SE framework was considered most appropriate to guide this review. One of the ecological frameworks which was considered was Bronfenbrenner’s ecological theory. It is a broad and encompassing theory of human development that takes into account, process, person, context and time [30–32]. However, the authors chose the Dahlgren and Whitehead’s SE model as it is more focused on social determinants of health and health inequalities [25,33].

In the Dahlgren and Whitehead’s SE framework, determinants of health are conceptualised as layers of influence and the complex interactions between these determinants [25,33]. The model depicts that people’s capacity to maintain their health is largely shaped by societal norms and networks, conditions in which they live and work, access to basic needs and the broader social, cultural, environmental and economic contexts [25,33]. Applying the SE framework, this narrative review takes a broad and comprehensive lens to fully explore the Nigerian health system as a core social institution [25,33,34].

The aim was to understand the capacity of existing health systems, policies, and support in Nigeria in meeting the health needs of PLWD with a special focus on people living with VI. The objectives were to:

1. Explore effectiveness of existing legal and policy frameworks in Nigeria on health service provision, social protection and rights of PLWD with a special focus on people living with VI.
2. Assess responsiveness, effectiveness and adequacy of the Nigerian public health system to address the healthcare needs of PLWD with a special focus on people living with VI.

In the next section, the rationale for conducting a narrative review is provided. We further discuss the analytical framework guiding this review: Dahlgren and Whitehead’s SE framework. This is followed by a delineation of the major databases that were searched to conduct data collection. The study then details the process of analysis of the retrieved data. These research processes are central to this review as they are necessary to answer the research questions.

## Materials and methods

### Rationale for narrative review approach

Narrative reviews are comprehensive descriptive synthesis of existing literature which deepens understanding [35–37]. They do not follow a prescribed methodology for the identification and selection of included sources [38–40] but usually provide inclusion criteria for choosing relevant cited resources on a subject matter [39]. Additionally, they lean towards qualitative interpretation of secondary data [41]. Overall, the rationale for choosing this form of review is that it is critical for the exploration of under-researched topics [42], the current status of issues and provides insights that are absent in the evidence base [43].

### Analytical framework

Dahlgren and Whitehead’s SE framework contains layers of influence which include: general socio-economic, cultural and environmental conditions, living and working conditions, social and community networks and individual lifestyle factors [25,33]. An additional layer consisting of age, sex and genetic makeup is included, however, Dahlgren and Whitehead acknowledge that these factors are fixed and that people have little control over them [25]. This model is centred on interactions whereby a person’s lifestyles are embedded within societal standards and networks, and in conditions in which people live and work, which are then connected to the broader socioeconomic and cultural environment [33].

Therefore, the model presents a holistic perspective of the major determinants of health [44]. This awareness will encourage a multisectoral collaboration which is considered by the authors as a more efficient way to address the healthcare needs of PLWD particularly people living with VI in Nigeria [44].

### Data collection

The layers of influence defined in the model informed a targeted literature search. Inclusion criteria focused on studies addressing disability, healthcare access, and Nigeria. Using the nomenclature of the Dahlgren and Whitehead’s SE framework, a comprehensive and iterative literature search was conducted in key databases such as Medline, Scopus, Embase and Google Scholar to access peer-reviewed literature on healthcare systems and policies for PLWD including people living with VI in Nigeria (quantitative and qualitative studies). Relevant grey literature such as government and non-governmental reports, policy and strategy documents were also sourced from international, intergovernmental, national government and non-governmental organisation (NGOs) websites such as World Bank, WHO and Nigeria Centre for Disease Control.

For the first layer of influence, the search focused on the Nigerian economy, policies and politics, disability, VI and poverty, institutional barriers and environmental factors particularly urbanisation and industrialisation. These included words such as disability and Nigerian economy, disability and poverty in Nigeria, VI, disability laws/policies in Nigeria, industrialisation/urbanisation in Nigeria. The literature search pertaining to the second layer centred on availability of work and education opportunities, health and health services and included keywords and phrases such as: disability and employment in Nigeria, people living with VI/blindness and employment in Nigeria, disability and education in Nigeria, VI/blindness and education in Nigeria, Nigerian health/healthcare system, epidemiological transition and Nigerian health insurance.

The third layer related to factors such as societal perceptions, attitudes and beliefs about disability/VI in Nigeria such as instances of discrimination, exclusion and stigma. The search employed phrases such as sociocultural aspects of disability/blindness/VI, stigma and VI, disability/VI and discrimination in Nigeria, financial exclusion and disability. These search terms are exemplars as one of the reviewers (ND) iteratively interchanged, combined and introduced new search terms to widen the scope of the search.

To ensure a comprehensive search, reference lists of relevant literature were also searched [45]. Additionally, no date limits were applied. From the search results, relevant literature in English were reviewed spanning a wide array of literature. The literature search was limited to only studies written in English or that could be translated to English as this was the official language familiar to all the authors. Also note that the study did not assess the quality of the included studies as this is generally not a requirement for narrative reviews [46].

### Data analysis

One reviewer (ND) conducted the literature search, identified and selected relevant documents in line with the objectives and the SE framework. The other reviewers (LD, HM, ZS and BE) revised and crosschecked the processes. After reading and selecting documents, relevant information was extracted. Data extraction was largely deductive driven by SE framework but with a degree of openness for unanticipated themes to emerge (inductive coding). Contents of retrieved literature were thematically and manually coded according to the layers of influence of the SE framework and then synthesised to produce a situation analysis. One reviewer (ND) conducted preliminary analysis of selected documents while the other members of the review team (LD, HM, ZS and BE) reviewed the analytical processes. The review team additionally had several meetings to discuss the review and resolve issues. Thematic saturation was reached when no additional themes emerged from data [47,48].

## Results

Findings are presented starting with an account of the political context followed by the economic and sociocultural contexts. This is followed by an account of the health system and policy contexts. Finally, the environmental context is presented. Specific factors identified were mapped on to the SE framework. Table 1 shows a summary of themes, subthemes, subcategories and corresponding layers of influence of the Dahlgren and Whitehead’s SE model.

**Table 1.** A summary of themes, subthemes, subcategories, and corresponding layers of influence of the Dahlgren and Whitehead’s socioecological model. Source: Adapted from Dahlgren and Whitehead, 1991 [25].

No	Overarching Layers of Influence Socioecological Model	
1.	**General socioeconomic, cultural and environmental conditions**	
	**Themes**	**Subthemes**
a.	Political context	Legal and policy support for anti-discrimination
b.	Economic context	Economic crisis
		Lack of inclusive education and unemployment
c.	Environmental context	Urbanisation
		Industrialisation
2.	**Living and working conditions**	
a.	Health system and policy contexts	Health profile
		Health system
		Health policies
		Health sector challenges● Corruption● Underfunding● Lack of cutting edge medical equipment● Short supply of healthcare workforce● Counterfeit drugs● Wastage● Ineffective leadership● Inaccessible medical information and infrastructure● Negative attitudes and behaviours of healthcare workers
3.	**Social and community networks**	
a.	Social and cultural contexts	Stigma and discrimination
		Marginalisation of PLWD from the mainstream financial system

Owing to inherent interconnections and, in some cases, no clear distinctions between layers of influence, the results are not entirely arranged by the layers of influence of the SE model. After analysing results mapped to the layers of influence, this section culminates with the thematic synthesis of the results. The different contexts do not operate in isolation hence, they need to be observed synthetically. Through this thematic synthesis, the authors observed a reciprocal influence which is described in depth after the Results section.

### Political context

This section presents the political landscape and the Nigerian government’s role in the formulation and enforcement of policies for PLWD and people living with VI. We argue that non-enforcement of existing anti-discrimination laws and policies negatively impact the health and wellbeing of PLWD. The political context section aligns with the outermost layer of influence of Dahlgren and Whitehead’s SE model which refers to the major structural environment as prevailing influences that mediate population health [25,33].

Federalism was introduced in Nigeria during British colonisation and has been the foundation of the system of government evolving from three regions in 1954–63 to the present day 36 states [49]. In Nigerian federalism, powers and resources are shared between the federal government, 36 federating units and 774 local government areas (LGAs) [49,50]. However, data shows that the federal allocation parameter for determining health needs is grounded in inputs such as the number of healthcare staff and hospital beds [51]. This has been cited as a flawed premise because richer states have greater number of healthcare workers (HCWs) and hospital beds [51]. Hence, some states may be more preoccupied with increasing these facilities to attract increased allocation rather than improving health outcomes and addressing inequalities [51]. Revenue allocation under Nigerian federalism thus reinforces inequitable distribution of health resources [51,52].

#### Legal and policy support for anti-discrimination

In 1993, the Nigerian government promulgated the Nigerians with Disability Decree [53]. The decree aimed to provide coherent and comprehensive security and protection backed by law for Nigerian PLWD and the establishment of rules for the enforcement of rights and privileges [53]. However, this decree was not implemented by successive governments [15]. There is no evidence that this decree was gazetted or included in the 2004 federation laws [54].

The main issue with the decree pertained to enforcement [55]. The Act stated that a National Commission for PLWD had been established [53]. The Commission was generally expected to engage in promoting the welfare of PLWD and to coordinate affairs between the PLWD and the government to eliminate sociocultural practices likely to subject PLWD to discrimination and dehumanisation [55]. However, lack of funding, staffing and limited ability to request recruitment data from employers meant that it could not initiate legal proceedings against alleged defaulters of the Act [55]. As a result, the Commission was ineffectual and ended before it could operate [55].

Furthermore, the decree contained a section directing every public health establishment to give medical and health services at no cost to PLWD [56]. Hence, the non-implementation of this Act deprived the PLWD of free medical services which could have pushed them into making high out-of-pocket (OOP) healthcare payments. This is particularly significant when considering that PLWD have increased risk of hospitalisation [6]. Research revealed that PLWD in Nigeria consistently had higher total OOP spending, healthcare expenses, OOP payment burden and greater risk of catastrophic health expenditure than the participants living without disabilities [57]. Catastrophic health expenditure can deplete the finances of PLWD resulting in poverty which can restrict further access to healthcare services.

Similarly, in 2010, the Nigerian government ratified the UN Convention on the Rights of Persons with Disabilities (UNCRPD) [15,58] however, no meaningful program was implemented for PLWD [15]. This was confirmed in a research which showed that despite the ratification of UNCRPD, there was lack of evidence to substantiate the implementation of the provisions of this policy [59]. Additionally, the study revealed that PLWD in Nigeria still faced disabling barriers in accessing different essential services due to absence of legal frameworks for the implementation of policies [59].

In 2019, the Discrimination against Persons with Disabilities (Prohibition) Act 2018 was signed into law for PLWD to be wholly integrated into the society [60]. However, again the law is not yet domesticated in the majority of Nigerian states [60]. This Act made provisions for equal opportunities in education, transportation, employment, health and additional areas for PLWD [61]. Nevertheless, according to UN Nigeria, there has been no significant progress towards attaining the level of equity mandated by the UNCRPD [61]. Findings from their research revealed implementation gaps and showed that the employment quota reserved for PLWD did not extend to the private sector hence this provision was weak and vague [61]. Additional problems included failure to mandate employers to make the work environment accessible and to provide digital accessibility and assistive technology for PLWD [61].

Inadequate and non-implementation of laws and policies can be traced to poor implementation strategies, insufficient budgeting, attitudinal barriers, low awareness of disability rights, lack of political will and lack of proper monitoring and evaluation [61]. As of 2023, little has been carried out concerning the implementation of these laws [6]. The Nigerian government’s reluctance toward the full domestication of the UNCRPD poses a significant hindrance to the adequate protection of PLWD [56]. This failure undermines effective protections against dehumanising and inhumane treatment, physical abuse, mental assault, neglect and discriminatory acts bordering on health services, transportation, employment and communication [56]. Table 2 shows the timelines of the anti-discrimination acts to date and corresponding implementation gaps.

**Table 2.** Timelines of the anti-discrimination acts to date and corresponding implementation gaps.

No	Year	Anti-discrimination Acts	Implementation gaps
1.	1993	Nigerians with Disability Decree	The National Commission which was set up to implement the Act was inadequately funded and staffed hence was ineffectual. This negatively impacted on the implementation of the Act [36].
2.	2010	United Nations Convention on the Rights of PLWD (Ratified)	PLWDs in Nigeria continue to face disabling barriers in trying to access various essential services due to absence of legal frameworks [38].
3.	2019	Discrimination against Persons with Disabilities (Prohibition) Act	The Act has not been domesticated in majority of the states [39]. Additionally, 5% employment quota reserved for PLWDs did not extend to private sectors resulting in a weak and vague provision in the Act [40]. Also, there was failure to mandate employers to make the work environment accessible and to provide digital accessibility and assistive technology [40].

#### Economic context

This section presents an overview of the Nigerian economy and its role as a determinant of healthcare access and health outcomes for PLWD and people living with VI. Education and employment are also analysed as contextual components that present equalising opportunities for the economic empowerment of PLWD and people living with VI in Nigeria. The economic context aligns with the outermost layer of influence in the SE model where socioeconomic factors shape population health [33]. This section focused on the missed opportunities for PLWD in Nigeria to be economically empowered.

##### Economic crisis

Nigeria is currently undergoing its worst economic crisis [62] with a record-high inflation which peaked at 40.9% for food, and 34.2% for every item in 2024 [63]. This is driven by fuel subsidy removal, increases in prices for food and energy, depreciation of the naira, and unchecked financial policies [62–70]. In the last decade, Nigeria’s gross domestic product (GDP) per capita income fell from $2,720 in 2015 to $835.49 in 2025 making it the lowest level throughout Nigeria’s documented history [71,72]. An additional 10 million Nigerians have been driven into poverty in 2023 as the living wage has not kept up with inflation [73] combined with a shortage of employment opportunities [70]. An estimated 38.9% of Nigerians lived below the international extreme poverty line of $2.15 a day in 2023 [73]. Furthermore, estimates of 69.8% and 91.9% of Nigerians lived below the lower middle-income poverty line of $3.65 and upper middle-income poverty line of $6.85 in 2023, respectively [73]. Yet, poverty is among the determinants of health service utilisation and outcomes [15].

Poverty and disability drive consequences including reduced access to health services, education, resources, opportunities and employment [74–76]. Approximately, nine in every ten PLWD in Nigeria live below the poverty line [74,77]. At household level, Nigerian families with at least one person living with disability suffer more monetary deprivation and multidimensional poverty than families without a person living with disability [78]. There is also disparity in multidimensional poverty with higher functional difficulty [79]. Hanass-Hancock *et al.’s* report showed that people who experienced many functional difficulties had higher percentages of multidimensional poverty at 81.3% compared to those with lesser and no functional difficulty which stood at 56.2% and 56%, respectively [79].

The link between poverty and blindness has been established in Nigeria [80]. A National survey reported poor households with higher prevalence of blindness and VI [80]. Additionally, a direct association has been established between severity of VI and the severity of poverty [28]. Hence, affordability and accessibility of healthcare may be challenging for people living with VI in Nigeria [28]. Another illustration of the challenges with economic access is that, in securing the funds required for OOP healthcare payments, people living with VI are often driven into debt [81]. This has been termed the medical poverty trap [82]. VI can thus be considered as condemning people in Nigeria to economic hardship/poverty and dislocation from services that might otherwise maintain quality of life and participation in education and employment.

### Lack of inclusive education and unemployment

Data showed that 95.5% of children living with disabilities were out of school in Nigeria as at 2020 [83]. Yet education is primarily the tool through which many PLWD can escape poverty and be full contributing members in the society [84]. To economically empower PLWD, they need to be given opportunities and an enabling environment to earn a living to support themselves [84]. However, the poor access of PLWD to qualitative and functionally inclusive education is among the factors behind their poor capacity to take up gainful employment [85].

For people living with VI, education, particularly tertiary education, boosted competitive employment [86]. Nevertheless, data revealed that people living with VI had the lowest percentage of literacy and were more likely to be previously employed compared to people living with other forms of physical disabilities [87]. Given the precariously low socioeconomic status of many people living with VI, it may be challenging to afford specialised educational materials [88]. This highlights a pervasive lack of opportunities for people living with VI to acquire the highest level of education possible [88]. The implication is further alienation of people living with VI from education and resultant missed opportunities to economically empower themselves to become financially independent and make meaningful contributions to the society.

Interventions at this layer of influence of the SE model usually revolve around applying economic strategies that will factor in the economic needs of PLWD in Nigeria [25]. This translates to policies that will secure good economic and environmental access to services such as inclusive education and employment [25].

### Social and cultural contexts

The social and cultural contexts section is represented at the third layer of the SE level where PLWD interact with members of the society who in turn influence them [33]. This section highlights societal barriers that serve to exclude PLWD and people living with VI from the society and access to essential services.

#### Stigma and discrimination

As well as a clinical and epidemiological concept, disability is a learned social role [89]. Stigma and discrimination against PLWD are endemic in Nigeria [1,15,90]. These manifest as negative attitudes among family members and society, misconceptions about disabilities, name-calling resulting in poor self-esteem, being isolated and depressed [1,91]. Particularly, people living with VI are often isolated due to the sighted persons’ beliefs that associating themselves with them reduces their dignity [92]. The literature demonstrated that PLWD in Nigeria have inadequate access to basic services and that their exclusion from key areas of socioeconomic activities are underpinned by deeply entrenched attitudinal barriers [1].

In 2020, the World Bank conducted research with PLWD in Nigeria on barriers encountered by PLWD, availability of disability-support services, funding, projects and existing policy and legal frameworks [1]. This research found that PLWD were subjected to persistent negative attitudes such as neglect, being considered as undeserving to live, loss of respect, rejection, derogatory labels and being regarded as useless [1]. The study also showed that PLWD experienced poor self-esteem, suicidal ideation, depression and isolation [1]. Another study revealed that negative attitudes to treatment procedures and perceptions on the root cause of eye diseases play significant roles in the reason people living with VI are not able to access health facilities [29]. Exclusion of people living with VI from healthcare endangers their health and wellbeing depriving them of equality of access to health services as their rights as Nigerian citizens.

**Figure 1.**
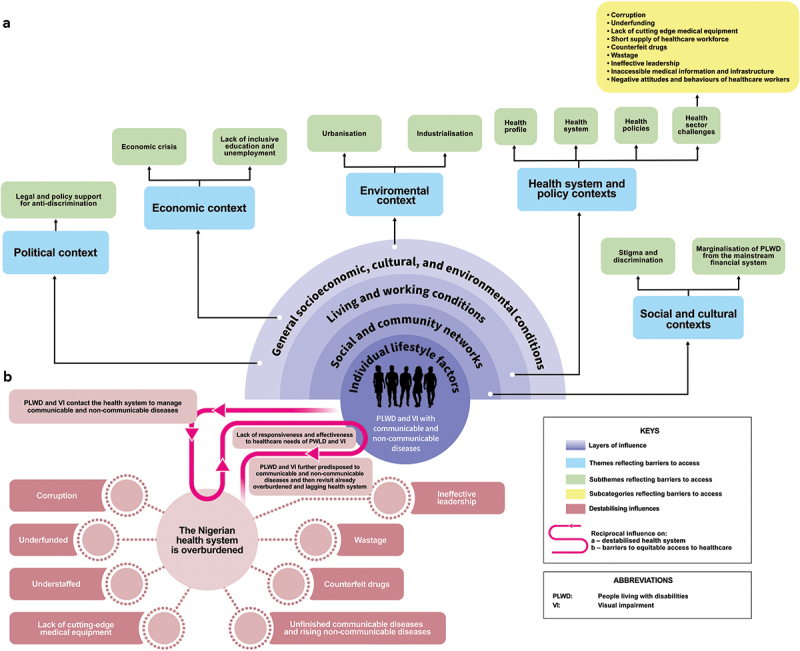
1a – Thematic analysis of barriers to access; 1b – Combined effects of destabilizing influences from the health sector resulting in the concept of reciprocal influence. Source: Adapted from Dahlgren and Whitehead, 1991 [25].

Additionally, earlier cited World Bank research found that health workers show negative attitudes towards PLWD especially with regards to sexual and reproductive health where they may disapprove of females living with disabilities engaging in sexual acts [1]. The study also found that if the PLWD are pregnant, the health workers may ridicule or blame them for it [1]. The health workers’ negative attitudes which can be due to ineffective leadership and lack of training, are reflections of those of the wider society who also discriminate against PLWD [1]. These can be interpreted as having the potential to dissuade PLWD from visiting hospitals or health centres when their services are needed. Non seeking of proper medical attention could aggravate any existing health issue or drive PLWD to the use of unorthodox medication [29]. With time this situation may worsen which may compel the PLWD to revisit the public health system where they may still encounter unaddressed negative attitudes, setting off a reciprocal influence (see Figure 1). The negative attitudes from health workers is discussed further in the Health and policy contexts section.

### Marginalisation of PLWD from the mainstream financial system

A qualitative study in Nigeria showed that PLWD experienced architectural barriers in accessing bank buildings [59]. Banks in a bid to strengthen security changed their entry points to include narrow mantrap portals which has made it impossible to allow people using wheelchairs to access banking halls [59] and financial services which may lead to financial exclusion. Another study on PLWD in Nigeria also revealed they are denied access to loan facilities on the premise of prejudice that PLWD have high tendencies of defaulting in loan repayment [93]. Moreover, the affinity for the culture of depriving the PLWD of the rights of inheritance complicates their miseries as they do not have the required collateral to secure loans [93]. This situation can perpetuate poverty as ineligibility to access loans could translate to not being able to start businesses that could be means of livelihood for PLWD.

According to Imandojemu *et al.*, many PLWD in Nigeria experience financial exclusion which shapes their aspirations and expectations [93]. Their analysis suggested that inaccessible structural layout of bank buildings is compounded by lack of assistive technology thereby preventing PLWD in Nigeria from accessing alternative formal financial services such as online banking [93]. The authors termed financial exclusion as a global wicked problem [93–95]. A wicked problem refers to a situation that is challenging or impossible to resolve due to its inherent complexity and interconnectedness [94,95]. This implies that the problem is dynamic, multisystemic and multidimensional [94,95]. Therefore, it may defy a lone solution rather, a multifaceted approach may be needed to mitigate it [94,95]. Another study revealed that ATM machines did not have speech prompts to aid people living with VI in carrying out financial transactions and that online banking websites were inaccessible [96].

The issue appears to be society’s unwillingness to make accommodations and equalising situations to address barriers in organisations and the environment [97]. When disabling environments exist and accommodations for PLWD are lacking, it shows a lack of knowledge, prejudice-based beliefs and discrimination [91,97]. These result in and enable the exclusion of PLWD from critical research and development of interventions and policies [97]. These then negatively impact on creation of services that are accessible, available and accountable which are central to the existence of PLWD as thriving members of the society [97].

Policies at this layer of influence of the SE model which are targeted at addressing the negative attitudes and beliefs towards PLWD include strengthening social and community support for them and their families [25]. Additionally, programmes aimed at creating awareness around educating the society against imbibing and acting on misconceptions about PLWD in general would be beneficial and impactful.

### Health system and policy contexts

This section corresponds to the second layer of influence of the SE model where an individual’s capacity to maintain their health is shaped by the conditions in which they live and work and access to essential goods and services such as healthcare [25,33]. Analysis centres on the Nigerian health profile and available public health facilities in Nigeria. This is followed by the analysis of the level of progress made towards achieving Universal Health Coverage (UHC) through health policies in Nigeria. The challenges besetting the Nigerian health system and its capacity to cater to the healthcare needs of PLWD and people living with VI were also analysed.

#### Health profile

Nigeria is a country in epidemiological transition [98]. Epidemiological transition theory was developed by Omran, who posited five stages in different societies [98]. These included pestilence and famine; receding pandemics; degenerative, stress and man-made diseases; declining cardiovascular disease, mortality, ageing and emerging diseases and; aspired quality of life with persistent inequalities [98]. Nigeria is currently in the third stage characterised as the age of triple health burden [98]. This comprises a double burden of disease where communicable and NCDs coexist in the Nigerian population [98–101]. Added to this double burden, most of the Nigerian primary healthcare (PHC) centres are faced with unavailability of essential NCDs drugs and key technologies for the treatment of major NCDs [102]. This is taking place in the face of rising chronic diseases [99] hence, triple health burden [98].

#### Health system

The three tiers of Nigerian government are concurrently responsible for providing healthcare in Nigeria [103]. PHC centres headed by LGAs, are the initial point of contact for preventive health while also promoting health and for the timely detection of disease or disability [103,104]. Secondary and tertiary health facilities give curative care, supervise and provide complementary services and get referrals from primary and secondary tiers where applicable [104]. The federal and state government head the tertiary and secondary healthcare facilities respectively [103]. Data shows that in 2013, there were 21,990 PHC centres while secondary and tertiary health facilities were 963 and 75 respectively [105].

#### Health policies

There is presently either a poor implementation of legal and policy frameworks for the protection and promotion of the rights of PLWD or they are non-existent in Nigeria [1,59,106,107]. The Nigerian government has a long history of enacting but not implementing or poorly implementing many of the anti-discrimination laws and policies for PLWD (see Table 2). In 1999, the Nigerian National Health Insurance Scheme (NHIS) came into existence, but its official launch was in 2005 for the provision of financial risk protection for Nigerians and the reduction of excessive burden of OOP payments on individuals and families [108]. Although the NHIS has been lauded as one of the ways countries can boost UHC [109], various inherent and systemic issues prevented the scheme from attaining UHC for every citizen [110]. Data indicates that NHIS has made significant progress in boosting access to medicines in Nigeria but this marginal boost in access is not adequate as the majority of Nigerians are unemployed and therefore not fully represented in the NHIS [110].

To salvage the existing health insurance scheme, the National Health Insurance Authority (NHIA) bill was signed into law in 2022 [111]. The objectives of the NHIA bill were the promotion, regulation and integration of health insurance schemes and to undertake any actions that will support the achievement of UHC for every Nigerian [112]. Significantly the NHIA Act established a Vulnerable Group Fund (VGF) to support the vulnerable population including the PLWD who find it difficult to pay for healthcare [113,114]. Although applauded as a ground-breaking initiative, data shows that the VGF is still inactive two years on because of financing barriers and incomplete implementation frameworks [115]. Furthermore, it is worthwhile to note that associations of PLWD in Nigeria have decried their not being actively involved in designing, planning, implementing and evaluating health policies, services and initiatives/projects [116]. Non-involvement of the PLWD for whom these health policies are enacted may be counterproductive as these policies may not have been based on the actual healthcare needs of the PLWD in Nigeria.

Additionally, there is no social program within the health system structured for the improvement of the equality of life of PLWD [15]. To describe this debacle, an analyst referred to PLWD as the visible but invisible in Nigeria [117]. However, the lack of health-related safety nets is deepening inequities in healthcare with a disproportionate impact on PLWD in Nigeria [115]. Data showed that PLWD encounter triple healthcare needs which include general health, disability-based health needs and disability-induced health needs [118–122]. Hence, PLWD usually need and utilise more healthcare services and incur greater healthcare costs than people without disabilities [76,116,118,119]. Yet PLWD are part of the poorest of the poor in Nigeria and therefore have diminished capacity to pay for needed health services [116,118,123]. The resultant impact is a deteriorating health outcome among PLWD [124] which is paradoxical considering that the premise of the UHC is being able to have unhindered access to comprehensive good-quality health services without financial difficulty [125].

##### Health sector challenges

The Nigerian health system is considered one of the most inefficient globally [15,126–129]. Nigeria ranked 142 out of 195 globally in the healthcare access and quality index in 2018 [128]. Different factors which have contributed to the deterioration of the Nigerian health system include: corruption [15,130–132], underfunding [126,133], inadequate supply of healthcare workforce, wastage, ineffective leadership [15], lack of medical equipment [15,133] and counterfeit drugs [15]. Two additional challenges which impact on the health of PLWD in the receipt of healthcare include: negative attitudes and behaviours of HCWs and inaccessible medical information and infrastructure. These factors are explained in detail in this section.

##### Corruption

In 2024, Nigeria was ranked the 40^th^ most corrupt nation globally [134] and the health system is especially susceptible to corruption [135]. Corruption cuts across various levels of the Nigerian health sector [136]. The major forms of corruption reported to have a high impact on patient outcomes include absenteeism, illegal payments in exchange for gaining access to care, diversion of patients to private hospitals and off-the-book charges to patients [136]. Another study reported up to 19 corrupt practices present in the Nigerian health system [130]. One of them is health financing corrupt practices which include extra billing of insured patients, hoarding of medications and falsely reporting stockouts of NHIS medications in health clinics [130]. Other corrupt practices include theft or diversion of money, medications and health supplies [130]. Furthermore, many health facilities are non-functioning due to practitioners’ absence from work or lateness [137]. It has been argued that, corruption is a major hindrance to high quality standard of healthcare and UHC in Nigeria [15,138]. Additionally, given that patients are compelled into paying bribes to obtain medications that are supposed to be free in some health facilities [137], PLWD may be disproportionately impacted. This is based on their diminished capacity to pay for essential health services [116,118,123].

##### Underfunding

The Nigerian health system is grossly underfunded [126,133] with budgetary allocation for 2024 at 4.6% of Nigerian’s GDP [139]. There is rather, a substantial dependence on international funding and NGOs to deliver most of the public health programs [126,140–142]. This implies that Nigeria may be at risk if the priorities of international funders shift [126]. A review reported that the Nigerian government healthcare budgets is persistently disconnected from the realities faced by patients which highlights significant challenges that keep impacting on healthcare service delivery [138]. Poor health financing elicits critical challenges acting as barriers to standard healthcare which include insufficient HCWs, inadequate infrastructure, ineffective leadership and high OOP payments [138]. For instance, underfunding of the health sector was linked to a high percentage of a study’s participants (85%) making OOP payments for healthcare services, depicting heavy reliance on personal funds to access healthcare [138]. This is potentially problematic for PLWD as they are more prone to OOP payment burden and greater risk of catastrophic health expenditure [57].

##### Lack of cutting-edge medical equipment

In Nigeria, accessing adequate healthcare equipment is challenging [143] and where they are available, some of them are antiquated [58]. Lack of medical equipment have been attributed to poor budgetary allocation to the health system [143]. As at April 2020, there were only 169 ventilators serving the entire Nigerian nation at the ratio of one ventilator to 1,266,440 persons [144]. Due to shortages of basic medical equipment in Nigeria, improvisation has become a persistent feature in the Nigerian health system [143]. A study showed that 31.9% and 22.78% of 2,480 Nigerian health facilities did not have sphygmomanometers and functional stethoscopes respectively [145]. Additionally, it has been reported that PLWD face barriers in access to public health infrastructure [58]. For instance, existing weighing scales and mammography screening equipment are designed for the patient to stand thereby making it inaccessible to PLWD who use wheelchairs [58].

##### Short supply of healthcare workforce

Brain drain of the healthcare workforce in Nigeria is a lingering issue [133,146]. The persistent migration of HCWs to other countries has reduced the Nigerian healthcare workforce to a critical level [147]. Consequently, Nigeria is experiencing shortage of medical professionals with approximately 35,000 doctors serving the Nigerian population despite needing 237,000 doctors as stipulated by WHO [133]. A survey of Nigerian medical doctors in 2017 showed that common reasons for emigration include better: quality of life, remuneration, workplace and facilities, career and professional advancement [146,147]. Continued brain drain of HCWs pose risks to the health of Nigerians particularly PLWD as they often require specialist healthcare services [58].

##### Counterfeit drugs

Nigeria is considered the most susceptible target market for counterfeit pharmaceuticals [148]. Therefore, counterfeiting of drugs is a public health menace in Nigeria [149]. In a study involving six sub-Saharan African countries, Nigeria had the highest incidence of failing samples of anti-malarial drugs at 63.9% [150]. The study reported that the possibility of getting substandard antimalarial drug was higher than the possibility of being treated with anti-malarial of internationally set standard of quality [150]. For instance, a Nigerian study revealed that Chlorine phosphate formulations’ failure rates were: 70% (20/29) for capsules, 100% (20/20) for syrups, 94% (17/18) for tablets and 93% (14/15) for injections [151].

Continued counterfeiting of drugs in Nigeria has been linked to persistent poor drug regulations and law enforcement, parallel trade, large market demand, complexity of the pharmaceutical supply chains, premium cost of medications [148,152] and bribery and corruption [148,153]. However, counterfeit drugs usurps the right of Nigerians to safe, effective and good quality drugs [153]. They can result in drain of finances, treatment failure, complications and death [137,148,153]. Counterfeit drugs hold severe health implications for people living with PLWD and VI. A typical example is the case of glaucoma which is an eye condition that can cause preventable blindness. However, the progression of VI from mild to severe can be halted using anti-glaucoma eye drops. Therefore fake anti-glaucoma medications could result in unabated vision loss culminating in total blindness for the affected persons thereby increasing the level of disability.

##### Wastage

Wasteful health spending mostly accounts for the gap between public health investment and outcome [154,155]. Studies have reported that healthcare service delivery in Nigeria is usually characterised by mismanagement which impacts on coverage and quality of healthcare services [15,145]. For example, many PHC centers in Nigeria built by the Federal Government at an estimated cost of $1.4 billion USD have remained unused despite a pressing need for these health facilities [156]. This has been traced to improper liaison and linkages with LGAs and states of location [156]. According to Balogun, PLWD are shortchanged when resources are in limited supply or wasted [15]. The impact of wastage inflates healthcare expenditures and lowers access to essential healthcare for PLWD [15].

##### Ineffective leadership

Poor leadership has stunted the development of the Nigerian health system [157]. Ineffective leadership at different levels of the health system is suggested to be the driver of the multifaceted challenges in the health sector [15]. For instance, one of the key problems to effective emergencies response include absence of LGA level leadership for local response [158]. Additionally, studies have reported that healthcare leadership and management challenges were shown to be the most common and important causes of strikes by HCWs in Nigeria [159–161]. The consequences of strikes on patient care are severe and they include disruption of patient care, worsening financial burden, increased morbidity and mortality, particularly for people living in poverty [161] such as PLWD. Conclusively, it has been reported that the lack of performance of the Nigerian health system can be attributed to government’s poor leadership role in health [162].

##### Negative attitudes and behaviours of healthcare workers

Evidence suggested that the relationships between HCWs and PLWD within health facilities in Nigeria have been less than satisfactory [1,163,164]. Data revealed that PLWD were subjected to negative attitudes especially with regards to sexual and reproductive health [1]. Furthermore, instances of humiliation, abuse and inequality towards PLWD in Nigeria by HCWs have been documented [163]. These negative attitudes and behaviours stem from ignorance in HCWs about the disease causing the disability, lack of skills and training in the inclusion of PLWD and undue pressure resulting from low doctor to patient ratio [1,163,165–167]. To address this, there should be training of HCWs to become inclusive health service providers and to create awareness regarding the right of PLWD to equitably access healthcare services [1]. Additionally, PLWD in Nigeria should also be sensitised to know that they have rights to health services [1].

##### Inaccessible medical information and infrastructure

For extended periods, PLWD in Nigeria have encountered many barriers in accessing healthcare services [168]. PLWD are reported to struggle to access hospital environment, equipment and health information [1,163,168]. In the qualitative research conducted by World Bank, participants narrated that health information and educational resources were not in accessible formats for PLWD in Nigeria [1]. Sign language interpreters were lacking in hospitals which meant that people living with hearing impairment had to rely on communication from family and friends thereby representing a breach of confidentiality [1]. Likewise, people living with VI had to depend on others to read the instructions pertaining to their medication and in some cases also compromising their right to confidentiality [1].

Furthermore, evidence suggests that medical information on signs and symptoms of diseases, treatment options including preventive measures is inadequate in accessible formats such as Braille, large prints and audio resources for ease of reading and comprehension [1,164,169]. According to Ayub and Rasaki, Nigerian patients with VI barely comprehend health trends and apply preventive measures to avoid disease [163]. Additionally, patients with VI may find it difficult to adhere to medications and other kinds of treatment, which can lead to poor health outcomes [169].

Lack of special accommodations for PLWD and people living with VI within hospital settings portrays the Nigerian health system as lacking in inclusive healthcare. Inaccessibility of health information and hospital environments may deter them from hospital visits or compel them to seek alternative means which could worsen their health conditions. The concept of how this situation can set off a reciprocal influence was discussed under the subtheme: Stigma and discrimination. Figure 1 gives a pictorial representation of this concept.

Although Nigeria made progress towards the UN’s Millenium Development Goals (MDGs) such as eradication of hunger, it did not meet any MDGs in 2015 with the exception of global partnerships for development [15,170]. In 2016, the UN launched the Sustainable Development Goals (SDGs) to eradicate poverty and inequality by 2030 [15,171,172]. Nigeria has made slight or no progress towards achieving the SDGs [15]. There is compelling unanimity amongst scholars and analysts that the Nigerian health system is in crisis and is in need of swift intervention that is if it is to meet the SDGs [15,173,174]. Analysts associate this with a lack of political will [15], a lack of effective progress evaluation, suboptimal insurance coverage at 3%, persistent industrial actions by HCWs [173] and ineffectively implemented strategies [174].

Policies at this layer of influence of the SE model are targeted at achieving sustainable structural changes [25].

### Environmental context

The environmental context is situated at the outmost layer of the SE framework as a mediator of population health in the overall society with prevailing environmental influences [25,33]. This section highlights the challenges presented by industrialisation and urbanisation in Nigeria.

Nigeria has undertaken various initiatives to drive industrialisation such as the Nigeria Industrial Revolution Plan, Manufacturing Sector Fund and other schemes [175]. With industrialisation comes urbanisation due to the migration of people to the cities in search of jobs created by industries and associated infrastructural development [176]. Evidence shows that industrial carbon emissions are driven by industrialisation and urbanisation in Nigeria [177,178] and they have adverse impacts on health [179]. Yet, carbon emissions in Nigeria are reported to be significant [179,180].

Climate change resulting from carbon emissions increases healthcare costs due to prohibitive costs of treating climate-sensitive diseases such as water and food borne diseases and heat stress [181]. Considering the higher likelihood of people living with VI to be affected by diseases [8,9], the negative impacts arising from industrialisation and urbanisation may be worse for this population. Their vulnerable circumstances may be compounded by poverty, low socioeconomic status, poor employability rate and low educational status which may place them in a disadvantaged situation to favourably combat the deleterious effects of greenhouse emissions.

Policies at this layer of influence of the SE model should focus on the health effects of various economic strategies which is critically important for any equitable health policy [25]. Additionally, there should be stricter measures on waste emissions from industries that have the capacity to endanger health.

## Thematic synthesis

Results were thematically grouped into five themes, eleven subthemes and nine subcategories all of which are intrinsically linked. Contextual factors which are grouped as themes converge and interact to prevent or restrict PLWD and people living with VI from equitable access to healthcare and other essential services. For instance, the weak or non-implementation of anti-discrimination laws and policies depicted under the political context can be responsible for the continued existence of inaccessible hospital environments, health information, hospital equipment and negative attitudes of HCWs towards PLWD under the theme: health system and policy contexts. In addition, the lack of inclusive education and unemployment can perpetuate poverty in PLWD under the theme: economic context and consequently limit PLWD’s capacity to afford prohibitive healthcare costs in the absence of health-related safety nets under the theme – Health system and policy contexts.

These interconnections between the themes may prove critical to finding cost effective solutions to the issues that the Nigerian health system is inundated with. By this the authors mean that addressing issues under a particular theme, could potentially resolve other challenges grouped under other themes. For instance strengthening implementation of anti-discrimination laws and policies, can have a ripple effect and resolve barriers that PLWD face with accessing health facilities and services equitably, unemployment, financial exclusion, lack of inclusive education, discrimination and stigma.

Furthermore, due to increased vulnerabilities of PLWD and people living with VI, they are more at risk of hospitalisation [6] thereby necessitating frequent contacts with an overburdened Nigerian health system. This stems from multifaceted challenges including understaffing, corruption [15], gross underfunding [126], and lack of medical equipment [15]. As such, lacks the requisite responsiveness and effectiveness necessary to adequately and equitably cater to the healthcare needs of PLWD and people living with VI. This sets off a reciprocal influence whereby PLWD and people living with VI are further predisposed to communicable and non-communicable diseases. Consequently, they revisit the overburdened health system thereby subjecting it to increased pressure. This phenomenon is referred to as the reciprocal influence resulting from an overburdened health system (Figure 1). This situation worsens over time and therefore, the intervention imperative is to act now. It is important to note that these are the interpretations of the authors based on this review’s extensive findings hence not yet empirically tested. In the absence of supporting longitudinal data, it remains a developed hypothesis.

## Discussion

This review provides a situation analysis of issues surrounding access for PLWD within the Nigerian health system with a special focus on people living with VI due to higher likelihood of reporting chronic and non-chronic conditions [8,9]. Applying an analytical framework, (Dahlgren and Whitehead’s SE model), we analysed peer reviewed and grey literature on the health system and policies for PLWD including people living with VI in Nigeria. Our findings demonstrate that while legal frameworks exist, they fail to translate into equitable service delivery, especially at the primary care level.

Existing research and evidence is relatively limited in scope and comprehensiveness and health system-focused [14–17]. For instance, one of the authors who previously reviewed the Nigerian health system suggested that the answers to Nigeria’s healthcare problems can only be resolved by healthcare providers in Nigeria, specifically ‘doctors’ [17]. On the contrary, our review has elaborately shown that the challenges faced by the health system go beyond the confines of the ‘doctors’ or even the health system itself. The use of a SE model as an analytical framework in this study ensured that the authors conducted this review with the understanding that many of the Nigerian health system’s challenges preventing inclusive healthcare emanate from the society within which it is situated. Hence, informed and viable solutions to these challenges need to be those that put into consideration the wider Nigerian societal contexts.

Our work therefore extends a health system analysis to a holistic, multi-sectoral synthesis, which demonstrated the extent of discrimination, stigma and failure to realise policy commitments and intentions. Our review aligns with, and extends, previous research in Nigeria by providing additional insights on how social, political and economic influences destabilise the health system and by extension the conditions of people’s lives. Additionally, our review reveals the self-sustaining nature of the Nigerian health system overburdened by diverse forces thereby highlighting implications for interventions (see Figure 1).

Our review resonates with the case study by Kuper et al. which acknowledged the extensive inequitable access to healthcare that PLWD in Zimbabwe (another low resource setting) face with worse health outcomes [182]. The Zimbabwean and Nigerian health systems share certain commonalities in the challenges they face in terms of lack of disability-inclusive health services for PLWD, OOP payments, exorbitant healthcare costs and underfunding [182]. The authors argued for the immediate prioritisation of disability-inclusive healthcare alongside strengthening of the Zimbabwean health systems which were inundated with widespread failures [182].

Furthermore, evidence showed that some other African countries were faced with similar problems in delivering disability-inclusive healthcare and services to PLWD [183]. Results from a research involving Sierra Leone, Zambia, Kenya and Uganda showed that despite policies, weak implementation resulted from lack of monitoring, issues with budget allocation and accountability, limiting the effectiveness of policies [183]. They concluded that disability and development gaps exist and attributed them to lack of focused legislation, persistent lack of planning and monitoring and policy gaps [183]. Implementation gaps were also compounded by lack of prioritisation, common prejudices and stigmas that overshadow essential needs, inequities that PLWD encounter and exclusion of PLWD and their organisations from administrative and political processes [183].

Despite long standing policy recognition and support by the Nigerian state for the enforcement of the rights of PLWD, actualisation of health policies through the health system (and other public systems such as social welfare) fails to realise stated policy ambitions and intentions towards PLWD. At present, there is a multiplicity of health policies in Nigeria. The government can focus on effectively implementing a select few health policies for PLWD and people living with VI that are low cost but high in gains thereby cutting down costs. Additionally, government can apply positive reinforcement by offering tax relief incentives to businesses which comply with modification of their buildings to become accessible to PLWD and people living with VI. However, the present economic crisis in Nigeria may impede actualisation of recommendations.

The authors observed an obvious lack of social welfare benefits for PLWD and people living with VI in this analysis. When there is no dependable governmental financial assistance policy, the majority of them are left without alternatives other than resorting to begging to survive and face hardship [184]. Properly designed and implemented social welfare schemes are capable of assisting PLWD to offset a lot of disability extra costs and support the inclusion of PLWD and people living with VI [185]. Hence, the authors’ recommendation to the Nigerian government is to make urgent provisions to initiate regular and substantive social welfare payments to PLWD and people living with VI in Nigeria while also giving additional special monetary allowances to people living with VI such as the Blind Persons Allowance as is practiced in high income countries such as the UK and the US [186,187]. These welfare benefits will assist in cushioning the effects of the economic downturn in Nigeria and improve access to essential services. How this is feasible in a context of federalism, poor governance, fiscal challenges and austerity measures is a critical consideration and cause for concern.

It is important to note that social protection for PLWD have been widely successful in some countries such as Vietnam. In 2018, Lena et al. conducted an extensive research in Vietnam to ascertain the disability inclusive social protection status [188]. They found that compulsory health insurance for households that had members living with disabilities significantly reduced healthcare expenditure when compared with non-recipients [188]. By 2021, the Vietnamese government made a decision to reform social protection [189]. The reform resulted in increased coverage for disability allowance which rose from 395,000 PLWD who benefited in 2011 to over 1 million in 2021 [189]. Critical success factors included government’s prioritisation of disability, enhanced accessibility to disability assessments and effective organisational structure at the grassroots focused on addressing the needs of PLWD [189].

In terms of tackling corruption, it is imperative to look beyond the health system and consider the wider economic, political and social contexts which shape practice [190]. Trying to tackle corruption among HCWs without giving careful consideration to working conditions and meagre pay (for instance) is not likely to be sustainable [191]. This translates to devising anti-corruption approaches focused on the development of locally tailored solutions instead of universal or up-scalable interventions [191]. If corruption is considered a systemic problem, then it is apparent that a multilevel approach is needed to tackle it – behaviourally and systemically [191].

Regarding healthcare funding, the percentage allocated for healthcare is extremely low and has not been able to effectively cater to the healthcare demands of the citizens particularly for PLWD and people living with VI. Hence, adequate funding for healthcare should be prioritised by the Nigerian government [15]. In reality, the actualisation of this recommendation appears bleak. This is due to the imposition of a 90-day halt of majority of the foreign aid to Nigeria by the US president [192]. Health programmes in Nigeria are funded by a large portion of the US foreign assistance [193]. It is unclear whether the fund freeze will continue after this stipulated period [192]. Although, the Nigerian government has stepped up to develop interim plans to fill the USAID funded health programmes gap [192], the sustainability of such programmes remains to be seen in the face of these austerity measures.

A pertinent finding was regarding negative attitudes of HCWs towards PLWD in Nigeria. This issue is not peculiar to the Nigerian health system. People living with VI in the US reported being recipients of negative attitudes by HCWs which manifested in the form of health staff touching them without asking for permission first and talking to their chaperones instead of speaking to them directly on matters pertaining to their health [194]. There were also assumptions that people living with VI were incompetent, consequently undermining their autonomy [194]. To curb these, the participants recommended that HCWs undergo training centred on the improvement of etiquette and communication, awareness of barriers to care and to dismantle ableist stereotypes and wrong perceptions [194]. These trainings can be replicated in the Nigerian context to support the HCWs in the uptake of disability-inclusive health practices.

Finally, the analysis illuminates the non-active involvement of PLWD in Nigeria in designing, planning, implementing and evaluating health policies, services and initiatives/projects [116]. The principle of participation by PLWD in these processes is aptly captured in the expression ‘nothing about us without us’ [195]. The essence is to ensure that the voices of PLWD and people living with VI in Nigeria are heard and programmes are structured in alignment with these voices which constitutes best practice.

The authors therefore recommend that the PLWD and people living with VI be actively involved at all levels in designing and implementing health policies and programmes in Nigeria. This approach has proven to be successful in some settings. For instance, a project was launched in 2018 for the improvement of healthcare access among the PLWD in Uruguay [189]. The participation of PLWD in designing and validating products not only constituted best practice but was also critical to the success of the program which led to the training of 300 HCWs in disability-inclusive healthcare using a human rights approach [189]. Additionally, the project led to the development of free accessible online health information and resources for PLWD in Uruguay [189].

### Strengths and limitations

This is the first study to date to comprehensively review the responsiveness and effectiveness of the Nigerian health system to address the healthcare needs of PLWD particularly focusing on people living with VI. This review makes significant contributions to disability health research by highlighting key issues acting as barriers to equitable access to essential services particularly healthcare in Nigeria. Our analysis is unique in its SE framing, synthesis of multisectoral evidence on how services are provided (or not) to support and enable PLWD and people living with VI to live full lives, participate in society and be economically productive and claim their right to health. The findings illuminate routes for intervention from which recommendations have been offered and considered in context.

Despite the strengths of this study, there are limitations to be considered and they include the following:

Although comprehensive, this review only analysed literature in English and sourced for literature from select databases and websites and may have omitted some relevant literature in other languages and sources respectively. This may have limited the scope of this review.

The review focused on only the Nigerian public health system. Some PLWD and people living with VI in Nigeria may receive healthcare from private health institutions and traditional settings. Hence, the findings in this review may not be representative of the healthcare access and delivery experiences of all the PLWD and people living with VI in Nigeria. Therefore, future research on the Nigerian health system can incorporate literature on private and traditional/alternative care systems and could also conduct a comparative analysis between these health systems.

Although inclusion of grey literature ensured an extensive search and lessened the risk of publication bias [196,197], it may have been potentially problematic. Grey literature is transitory and not broadly indexed, and may be challenging to source over time [198].

Use of deductive coding may have introduced confirmation bias [199]. However, collaborative participation of the review team limited this possibility. Additionally, the authors minimised the potential for missing relevant contents by using a combined approach (deductive and inductive coding).

Finally, this is a desk-based review as such no primary data were included. Primary data, however, are integral to health research as it gives people with lived experiences the opportunities to express their realities authentically. This often supports more tailored routes of intervention given that the research results emanated from the people experiencing the issues under study. Therefore, the authors recommend that future studies conduct lived experience research with people living with VI to gain firsthand accounts of their realities with accessing healthcare services in Nigeria. This can provide relevant empirical data that can form the basis for the formulation and implementation of disability-inclusive health policies in Nigeria.

## Conclusion

The Nigerian health system is in crisis and presently does not adequately address the healthcare needs of PLWD including people living with VI. For people living with VI in Nigeria to have a higher predisposition to diseases and still be of low socioeconomic status, suffer exclusion from health policies, healthcare services, socialisation, financial services and institutions, built environments and other essential services resulting in health inequalities is a wicked problem. Having established the health system as a core social institution, the study findings revealed routes of intervention that transcend the Nigerian health system to incorporate the wider social, cultural, economic and political landscape within which the health system is situated.

The analysis underscores the urgent need for systemic reform in Nigeria’s health system to ensure equitable access for PLWD including people living with VI. While legislative progress has been made, implementation remains inconsistent. Future research should explore the lived experiences of PLWD through qualitative designs and evaluate the effectiveness of pilot programs aimed at improving accessibility and inclusion.

## Data Availability

Data sharing is not applicable to this article as no data were created or analysed in this study.

## Abbreviations

GDP: Gross domestic product
HCWs: Healthcare workers
MDGs: Millenium Development Goals
NCDs: Non-communicable diseases
NGOs: Non-governmental organisations
NHIA: National Health Insurance Authority
NHIS: National Health Insurance Scheme
OOP: Out-of-pocket
PHC: Primary healthcare
PLWD: People living with disabilities
SDGs: Sustainable Development Goals
SE: Socioecological
UHC: Universal Health Coverage
UNCRPD: United Nations Convention on the Rights of PLWD
VI: Visual impairment
VGF: Vulnerable Group Fund
